# *Heterorhabditis bacteriophora* Excreted-Secreted Products Enable Infection by *Photorhabdus luminescens* Through Suppression of the Imd Pathway

**DOI:** 10.3389/fimmu.2019.02372

**Published:** 2019-10-04

**Authors:** Eric Kenney, John M. Hawdon, Damien O'Halloran, Ioannis Eleftherianos

**Affiliations:** ^1^Infection and Innate Immunity Lab, Department of Biological Sciences, George Washington University, Washington, DC, United States; ^2^Department of Microbiology, Immunology, and Tropical Medicine, George Washington University, Washington, DC, United States; ^3^Institute for Neuroscience, Department of Biological Sciences, George Washington University, Washington, DC, United States

**Keywords:** parasitic nematode, *Drosophila*, innate immunity, Imd pathway, *Heterorhabditis*, *Photorhabdus*

## Abstract

Upon entering the hemocoel of its insect host, the entomopathogenic nematode *Heterorhabditis bacteriophora* releases its symbiotic bacteria *Photorhabdus luminescens*, which is also a strong insect pathogen. *P. luminescens* is known to suppress the insect immune response independently following its release, but the nematode appears to enact its own immunosuppressive mechanisms during the earliest phases of an infection. *H. bacteriophora* was found to produce a unique set of excreted-secreted proteins in response to host hemolymph, and while basal secretions are immunogenic with regard to *Diptericin* expression through the Imd pathway, host-induced secretions suppress this expression to a level below that of controls in *Drosophila melanogaster*. This effect is consistent in adults, larvae, and isolated larval fat bodies, and the magnitude of suppression is dose-dependent. By reducing the expression of *Diptericin*, an antimicrobial peptide active against Gram-negative bacteria, the activated excreted-secreted products enable a more rapid propagation of *P. luminescens* that corresponds to more rapid host mortality. The identification and isolation of the specific proteins responsible for this suppression represents an exciting field of study with potential for enhancing the biocontrol of insect pests and treatment of diseases associated with excessive inflammation.

## Introduction

The early steps of a *Heterorhabditis bacteriophora* infection are well-described with regard to the physical actions of the parasite. Upon migration to a host, the majority of the infective juveniles (IJ) enter the insect through natural openings, although the IJ can generate tears in the intersegmental membrane to gain entry (1). Once the parasite enters the hemocoel environment, the nematode slowly releases, following a 30-min lag time, the bacterial endosymbiont *Photorhabdus luminescens* that it maintains as a secondary phase in its gut (2). When considering the molecular host-parasite interactions that determine the success of an infection after IJ entry, *Photorhabdus* often draws a substantial amount of interest due to its assortment of proteases and other factors that can suppress the insect immune response and lead to rapid death. However, it is crucial to recall that axenic nematodes are still capable of inciting insect mortality without their symbiont (3). Furthermore, numerous reports have shown that the immune-based neutralization of the nematode is possible. While IJs have been shown to evade encapsulation in *Tipula oleracea, Popillia japonica, and Cyclocephala borealis* (4, 5), the Colorado potato beetle *Leptinotarsa decemlineata* prevents IJ development through encapsulation, in which the process of hemocyte attachment to the parasite begins as quickly as 15 min after entry, a period comfortably preceding the release of bacteria (6). Generally, the degree of melanization and encapsulation of the IJ has been shown to correlate to the survival of the insect (7), so the nematode must to some degree fare for itself in terms of immune suppression during the early phase of an infection. Additionally, *Heterorhabditis* has a vested interest in promoting *Photorhabdus* survival, so some early IJ-based immune suppression may also be targeted toward developing a more hospitable hemolymph environment for its symbiont.

Much of the work centered on the entomopathogenic nematode infection process has used *Steinernema carpocapsae*. A pair of serine protease inhibitors from this nematode have been found to impair hemocyte aggregation, prevent clotting fibers from forming properly, inhibit the digestive enzymes of the host, and prevent the inclusion of melanin into clots formed in the hemolymph (8, 9). The bulk secreted proteins have also been found to be lethal when injected into *Drosophila melanogaster* adult flies (10), clearly indicating that the nematode plays a strong role in the molecular aspect of the infection aside from merely releasing its symbiotic bacteria. Less is known about the activity of the specific molecules produced by *H. bacteriophora*, but genome and transcriptome studies have predicted a variety of secreted factors (11, 12) and genes for a putative metalloprotease, enolase, and chitinase have been implicated in parasitism specifically (13). Genes for C-type lectin and catalase have also been found to be upregulated upon activation of the nematode, where the former is believed to play a role in immune evasion and the latter a role in protecting the parasite from free radicals. Both are expressed in other parasitic helminths, with the lectin being found in a range of nematodes from *Meloidogyne javanica* to *Ancylostoma ceylanicum* (14).

The molecular effects of an *H. bacteriophora* infection are likely the product of a collection of these effectors fulfilling a variety of roles, each of which is important for understanding the host-parasite relationship, but a number of practical applications await the identification of specific individual factors. Autoimmune disease, for instance, is believed to be exacerbated by the loss of natural associations with helminth parasites, and individual immunosuppressive factors isolated from nematodes could be effective treatments for conditions like Crohn's disease, asthma, or multiple sclerosis due to their specificity of action and tolerability (15). Entomopathogenic nematodes (EPNs) including *H. bacteriophora* are also currently used as biocontrol agents against insect pests (16), and manipulating these nematodes to make them more effective parasites could increase their efficacy. Other EPNs including *S. carpocapsae* are also viable options for biocontrol, but it is important to consider that not every EPN is as successful as others against a given host. When infecting the carob moth *Ectomyelois ceratoniae, S. carpocapsae* is dramatically more adept at overwhelming the host, with an LC_50_ of 2.02 IJs per larva as opposed to the 426.92 IJs required for the same activity by *H. bacteriophora* (17). When infecting the tomato leaf miner *Tuta absoluta*, however, *H. bacteriophora* is just as effective if not more so than *S. carpocapsae* (18). With this in mind, an optimal approach to developing strong biocontrol would not ignore either species.

Here we examine the immunosuppressive effects of *H. bacteriophora* bulk secretions on the *Drosophila melanogaster* immune system, and depict the degree to which this suppression compromises the insect with regard to susceptibility to a bacterial infection. Because the nematode's symbiont *P*. *luminescens* is such a strong pathogen, we hypothesize that the organisms have polarized each other's role in the infection and *H. bacteriophora* has become more specialized for immune suppression during the early phases of an infection for the benefit of the nematode as well as its symbiont.

## Materials and Methods

### Insect and Bacterial Strains

*Galleria mellonella* larvae were acquired from Petco and *Manduca sexta* from DBDPet. Fly stocks were maintained on a cornmeal-soy-based diet (Meidi laboratories) with added baker's yeast and incubated at 25°C on a 12-h day-night cycle. The *Drosophila melanogaster* lines used included *Oregon R* for *P. luminescens* survival experiments, phagocytosis assays, and gene expression analyses, *w*^1118^ for survival experiments with triple-concentrated ES product and *Escherichia coli* co-injections, *Rel*^*E*20^ for the *E. coli* co-injection assays, and the Diptericin(Dpt)-GFP line *T4202 (III)* for the transcriptional activation assay. Bacterial strains included *Photorhabdus luminescens* subspecies *laumondii*, strain TT01, the *E. coli* strain K12, and the RET16 derivative of the *Photorhabdus temperata* strain NC1. *Photorhabdus* strains were cultured on MacConkey Agar (Sigma) at 28°C for a period of 48 h at which point a single colony was used to inoculate an overnight liquid culture in 10 mL of Lysogeny Broth (LB) media (VWR) incubated at 28°C in a rotary shaker set to 220 rpm. *E. coli* was cultured in a similar fashion, but initial growth on agar was carried out on LB agar at 37°C overnight.

### Culturing Axenic *Heterorhabditis bacteriophora* Infective Juveniles

Infective juveniles of the rhabditid nematode *Heterorhabditis bacteriophora* strain TT01 were maintained axenically through propagation in *G. mellonella* larvae carrying well-established infections of RET16. To establish the infection, 1 mL of an overnight RET16 culture was centrifuged for 3 min at 13,000 × g, the supernatant discarded, and the pellet washed once with sterile phosphate buffered saline (PBS). The resulting bacterial suspension was centrifuged again and resuspended, at which point the suspension was diluted 1:10 with sterile PBS, to a final volume sufficient for the injection of 50 μL of bacterial solution into the desired number of 5th to 6th instar *G. mellonella* larvae. To perform larval injections, *G. mellonella* larvae were surface-sterilized by brief submersion in a 70% solution of ethanol. The larvae were placed on ice for a period of 20 min in a 100 × 15 mm petri dish furnished with moistened 90 mm filter paper. Injections were performed with a 1 mL tuberculin syringe and 22G needle inserted in the intersegmental region at as shallow an angle as possible. Larvae were left on ice for 5 min post-injection and then kept at room temperature for 1 week. Successful RET16 infection caused the larva to die and turn the brick red color typical of a *Photorhabdus* infection. Those that did not display the appropriate color were discarded. After 1 week, *H. bacteriophora* IJs were pelleted, surface sterilized with a 3% bleach solution for 5 min, and washed twice with sterile water prior to their liberal application onto the infected *G. mellonella* at a concentration of ~500 IJs per larva. This secondary infection was allowed to progress in the dark at room temperature for 8 days at which point the larvae were transferred to white traps for the collection of emerging IJs in autoclaved water supplemented with 0.01% Tween 20 (19). To confirm that the IJs were axenic, an aliquot of the surface-sterilized, putatively axenic IJs was used to infect *G. mellonella* larvae, and the larvae monitored for coloration indicative of an infection and support for the growth and reproduction of the IJs. IJs were considered axenic if they failed to produce red pigmentation in larvae or propagate successfully as compared to a surface-sterilized symbiotic IJ control.

### Preparation of Hemolymph From *Manduca sexta*

Approximately 500 μL of raw hemolymph was collected from each 5th instar *Manduca sexta* larvae. Prior to extraction, each larva was placed on ice for a period of 20 min. The area surrounding the posterior horn of the insect was treated with a 70% alcohol wipe just prior to the severing of the horn with microdissection scissors. This was performed directly over a 1.5 mL autoclaved microcentrifuge tube, as the release of hemolymph from the site of injury is rapid and immediate. To prevent melanization, an aliquot of 20 mM phenylthiourea dissolved in PBS was added to each aliquot of hemolymph to a final concentration of 0.33 mM. The extracted hemolymph was centrifuged for 5 min at 4000 × g and 500 μL of the resulting supernatant was added to 500 μL of ice-cold Ringer's buffer (100 mM NaCl, 1.8 mM KCl, 2 mM CaCl2, 1 mM MgCl2, and 5 mM HEPES adjusted to a pH of 6.9) in a separate sterile 1.5 mL microcentrifuge tube. For long-term storage, samples were frozen at −80°C. Before use, hemolymph was thawed on ice, diluted 1:1 in ice-cold Ringer's buffer, and filtered with a 0.45 μm syringe filter. Ampicillin and kanamycin were added to diluted hemolymph plasma solutions at concentrations of 100 and 50 μg/mL, respectively.

### Hemolymph Activation of Infective Juveniles and Isolation of Concentrated ES Products

Prior to activation, IJs were sedimented in aliquots of 200,000 and surface-sterilized with 3% commercial bleach in 10 mL of 0.01% Tween 20, resulting in a final hypochlorite concentration of 0.26%. Bleach-treated IJs were pelleted by centrifugation for 30 s at 1300 × g and washed twice with sterile Ringer's solution containing 0.01% Tween 20. After the second wash step, IJs were pelleted and resuspended in either 10 mL of the 25% hemolymph plasma solution (activated) or 10 mL of Ringer's-Tween (non-activated) containing antibiotics. The IJ suspensions were transferred to T75 tissue culture flasks, which were subsequently wrapped in foil and placed in a shaking incubator at 27°C and 200 RPM. Following a 20-h incubation, the IJs were transferred to 15 mL conical tubes, centrifuged, and washed 10 times with 10 mL of Ringer's-Tween 20 solution. Following the final wash, the IJs were resuspended in 10 mL of Ringer's solution without Tween 20. These tubes were wrapped in foil and returned to the incubator for 5-h at 27°C and 200 RPM to collect ES products. After incubation, the supernatants were removed and placed in a separate sterile 15 mL conical tube. The collected ES products were either stored at −80°C or immediately concentrated. To concentrate the collected products, ES products were filtered through a 0.2 μm low protein-binding syringe filter (Millex) and transferred to a new sterile 15 mL conical tube. Filtered products were added to a Vivaspin 6 tube (GE Healthcare) with a 3 kDa molecular weight cutoff, with aliquots of each treatment being added sequentially to the tube as sufficient volumes of solution cleared the filter. Concentration was allowed to continue until the volume of the retentate fell below 100 μL, at which point the solution was collected and supplemented with additional sterile Ringer's buffer to a final volume of 100 μL. For the triple-concentration of ES products, the same protocol was followed except that the ES products were initially distributed between two Vivaspin tubes, and the final 500 μL from each tube pooled and concentrated in a single tube until the volume was below 100 μL. ES concentrations were expressed as larval equivalents (LE/μL) by dividing the number of IJs used by the final volume of ES products.

### Protein Electrophoresis

Protein concentration of the ES products was quantified using a Pierce BCA Protein Assay Kit (Thermo Scientific) according to the manufacturer's instructions. For samples that produced a readable concentration of protein above the threshold sensitivity of the BCA assay, 6 μg of protein were loaded into a Novex WedgeWell 4–20% Tris-Glycine Gel (Invitrogen) following reduction in 50 mM DTT. For samples not producing a readable signal for protein concentration, the maximum volume was added to the gel. The final volume added to each well-included 26 μL of sample and water, 4 μL of the reducing agent, and 10 μL of Laemmli buffer. Protein size was demarcated with PageRuler Plus Prestained Protein Ladder (Thermo Scientific) and gels were stained with a Pierce Silver Stain for Mass Spectrometry kit (Thermo Scientific).

### Injection of *Drosophila melanogaster* Adults and Larvae

For survival and gene expression analyses, treatments were loaded into an oil-filled pulled glass capillary mounted on a Drummond Nanoject III Programmable Nanoliter Injector. Adult flies aged seven to 10 days were anesthetized with carbon dioxide and injected intramesothoracially with 69.0 nL of ES products or buffer, corresponding to 138 IJ equivalents of ES products, or 414 for triple-concentrated products. Injected flies were returned to vials containing instant *Drosophila* medium (Carolina Biological) and kept at 25°C on a 12-h day-night cycle. Flies injected for gene expression analysis at a 6-h time point were consistently injected in the late morning to alleviate effects attributable to natural variability arising from the circadian cycle. Wandering 3rd instar larvae were injected with 50.2 nL of ES products, representing ~100 IJ equivalents. Each insect was washed once with Ringer's solution upon removal from their original vial. Larvae were anesthetized with carbon dioxide for ~2–3 min before transfer to moist filter paper for injection. In order to ensure accurate, consistent injections, larvae were secured at the posterior end with forceps and injected at a shallow angle in an intersegmental region of the dorsal side of the abdomen to avoid damage to the organs or imaginal discs. Larvae were returned to a fresh petri dish furnished with filter paper moistened with Ringer's solution and incubated under the same conditions.

### qRT-PCR Analysis for Immune Gene Expression

At the indicated time points, five adult flies (three males and two females) or five larvae were collected in duplicate and total RNA was extracted using TRIzol reagent (Ambion, Life Technologies). Reverse transcription was carried out using a High-Capacity cDNA Reverse Transcription Kit (Applied Biosystems) and 1 μg of RNA template. The subsequent RT-PCR reactions were performed in a CFX96 Real-Time System, C1000 Thermal Cycler. The reactions themselves consisted of 10 μL of GreenLink No-ROX qPCR Mix (BioLink), 40 ng of cDNA template, forward and reverse primers at a final concentration of 200 nM, and ultrapure water to a final volume of 20 μL. Cycle conditions were as follows: 95°C for 2 min, 40 repetitions of 95°C for 15 s followed by 61°C for 30 s, and then one round of 95°C for 15 s, 65°C for 5 s, and finally 95°C for 5 s. The primer sets used for amplification included those for *Diptericin* (F: 5′ GCTGCGCAATCGCTTCTACT 3′; R: 5′ TGGTGGAGTTGGGCTTCATG 3′), *Cecropin* (F: 5′ TCTTCGTTTTCGTCGCTCTC 3′; R: 5′ CTTGTTGAGCGATTCCCAGT 3′), *Drosomycin* (F: 5′ GACTTGTTCGCCCTCTTCG 3′; R: 5′ CTTGCACACACGACGACAG 3′), *mcf1* (F: 5′ AAGGAGGTCAATGCTCGCTAC 3′; R: 5′ GACACAACTAATCTGCCGTTCTC 3′), *P. luminescens* 16S rRNA (F: 5′ ACAGAGTTGGATCTTGACGTTACCC 3′; R: 5′ AATCTTGTTTGCTCCCCACGCTT 3′), and *rp49* (F: 5′ GATGACCATCCGCCCAGCA 3′; R: 5′ CGGACCGACAGCTGCTTGGC 3′). Fold change was calculated using the 2^-ΔΔC^ method (20, 21) with all values being normalized to *rp49*. Graphs show fold change for each treatment over 0-h expression and error bars represent standard error applied to ΔΔCt values prior to conversion to a log scale. Statistical analysis was performed with a one-way ANOVA for ΔΔCt values accumulated from three biological replicates with two technical replicates each.

### Fat Body Dissection and Imaging for ES-Injected Dpt-GFP Larvae

Larvae of the Dpt-GFP *Drosophila* line were injected with 50.2 nL of non-activated or activated ES products according to the aforementioned injection protocol. Following the 6-h incubation period, the fat body was dissected out of the insect, but left attached to the body while the gut was removed completely. Tissues were fixed in PBT (PBS containing 0.2% Triton X-1000) with 4% paraformaldehyde for a period of 30 min. Three 10-min washes in PBT were performed followed by a 30-min incubation with TRITC (Molecular Probes) diluted 1:100 in PBS. After washing once with PBS, the fat body tissues were removed from the insect carcass, cut into pieces small enough to lie flat on a slide, and mounted with Antifade mounting medium with DAPI (Molecular Probes). Images were acquired with a Zeiss LSM 510 confocal microscope and corrected total fluorescence measurements were processed for isolated green channels using ImageJ software. Ten images were analyzed per treatment for each trial.

### Co-injection of ES Products With *Escherichia coli* and *Photorhabdus luminescens*

Co-injection solutions were prepared by mixing ES products and bacterial suspensions such that each injection contained 310 larval equivalents of ES products and either ~ 8 × 10^4^ CFUs of *E. coli* or 50 CFUs of *P. luminescens*. This was achieved by diluting cultures of *E. coli* (OD600 of 3.0) or *P. luminescens* (OD600 of 0.4) 1:4 in the triple-concentrated ES products. All solutions were mixed immediately prior to use and injected using the same injection protocol. For consistency, control treatments were likewise comprised of PBS diluted 1:4 in Ringer's solution.

### Quantification of Phagocytic Activity

Phagocytic activity was assessed by measuring fluorescence following the injection of pHrodo Red *E. coli* BioParticles Conjugate for Phagocytosis (Molecular Probes). A 4 mg/mL suspension of pHrodo particles was diluted 1:4 in ES products such that each co-injection contained 310 larval equivalents in a 1 mg/mL solution of pHrodo particles. Upon injection, flies were incubated at 25°C for 1 h at which time the dorsal side of the abdomen associated with the pericardial nephrocytes was imaged using a Nikon ECLIPSE N*i* microscope at 10x magnification with a Zyla (ANDOR) 5.5 camera. Corrected total fluorescence was measured using ImageJ software.

### Statistical Analysis

All statistical analyses were performed using GraphPad Prism 5 software. Gene expression analyses and CTF measurements for the phagocytosis assay were compared using a one-way ANOVA and Bonferroni multiple comparisons test to determine differences between specific treatments. Significance for CTF measurements for the Dpt-GFP assay was determined with a Student's *t*-test, and survival curves were assessed using a Log-Rank (Mantel-Cox) test. All analyses were performed on data accumulated through three independent experiments.

## Results

### Exposure of *Heterorhabditis bacteriophora* Infective Juveniles (IJs) to Host Hemolymph Induces the Secretion of Unique Proteins

**Figure 1:**
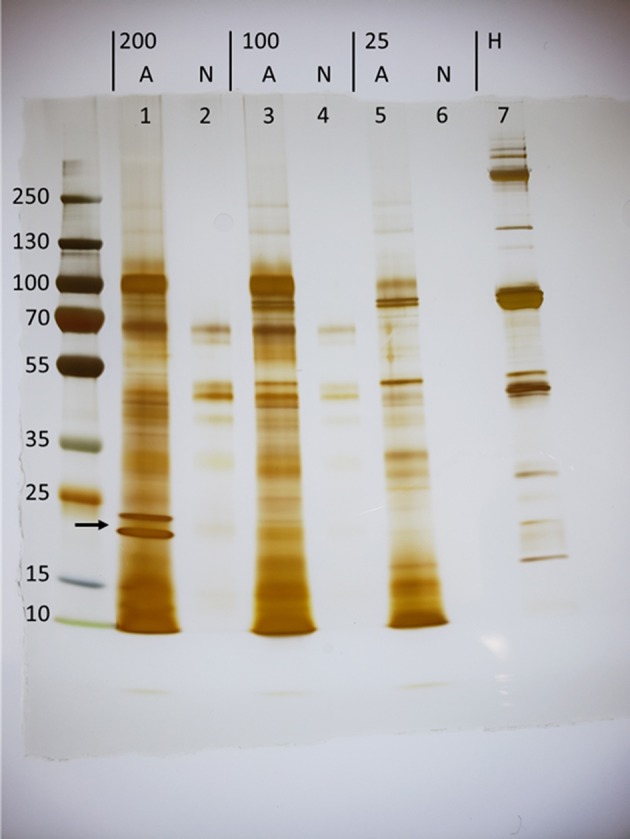
Exposure of *Heterorhabditis bacteriophora* infective juveniles (IJs) to host hemolymph induces the secretion of unique proteins (arrow). Concentrated ES products were loaded for groups of 200,000 (200), 100,000 (100), and 25,000 (25) nematodes that were either activated (A) in *Manduca sexta* hemolymph or left non-activated (N) in Ringer's buffer. Activation took place over a period of 20 h, at which point IJs were washed, transferred to fresh ringers, and incubated for 5 h to collect ES products. Lanes 1,3, and 7 carry 6 μg of protein while all others were loaded with a maximum volume, as protein could not be detected using the BCA assay.

To investigate the proteins secreted in response to host stimulus, groups of 200, 100, or 25 thousand (k) *H. bacteriophora* IJs were activated as previously described (11). IJs were activated for 20 h by incubation in 25% *Manduca sexta* hemolymph diluted in Ringer's buffer, washed several times, and transferred into fresh Ringer's buffer without hemolymph to collect ES products. This activation time point was selected based on preliminary experiments in order to optimize as closely as possible an *in vitro* activation that may be only minimally informed by knowledge of *in vivo* activation kinetics. Filtered collection buffer was subsequently concentrated by ultrafiltration through a 3 kDa cutoff membrane, which restricts the analysis to proteins rather than small molecules. Activated batches of 200, 100, and 25 k IJs yielded 286, 216, and 39 ng/μL of protein, respectively, whereas protein was undetectable in ES products collected from similar numbers of non-activated IJs incubated in Ringer's throughout. To visualize proteins present in the ES products, 6 μg of activated ES products were separated by SDS-PAGE and silver stained (Figure 1). The maximum volume of non-activated ES products (26 μl) were used because protein was undetectable. A comparison of the lanes shows that certain species of protein are unique to the ES products of activated nematodes, with two conspicuous examples in the activated 200 K lane at estimated molecular weights of 21.2 and 18.9 kDa. Importantly, these proteins are absent from the *M. sexta* hemolymph, confirming that the extensive washes following the 20-h incubation removed residual hemolymph. This indicates that *H. bacteriophora* IJs specifically release a unique suite of proteins in response to hemolymph exposure.

### *Heterorhabditis bacteriophora* Nematode Excreted/Secreted (ES) Products Elicit Differential *Diptericin* Responses That Are Consistent Across *Drosophila melanogaster* Life Stages

**Figure 2:**
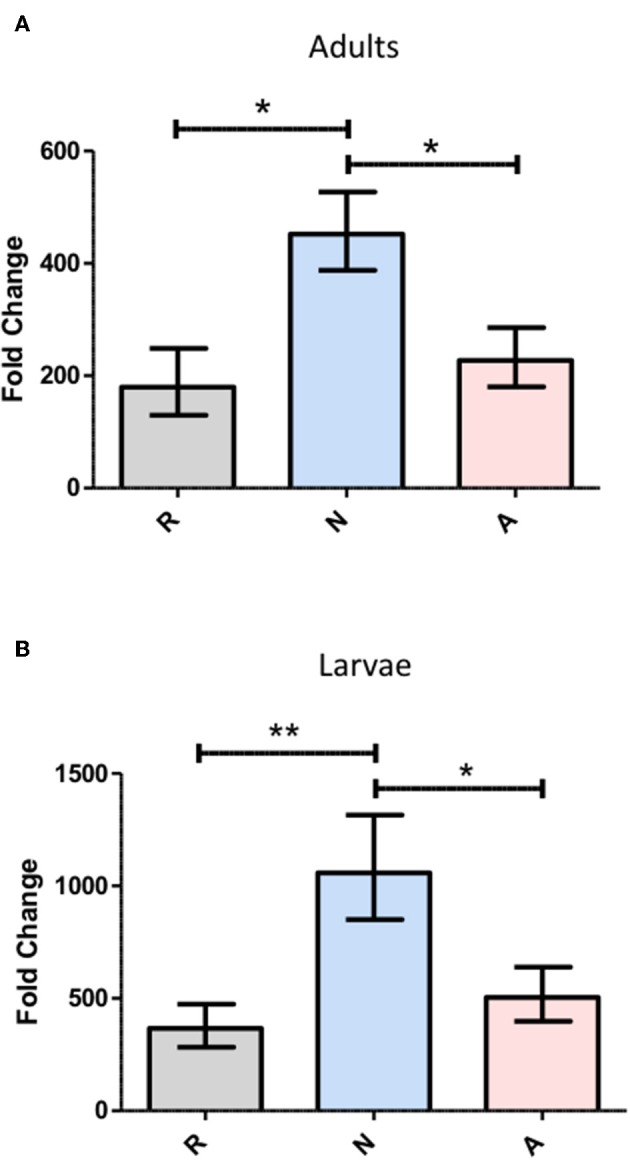
*Heterorhabditis bacteriophora* nematode Excreted/Secreted (ES) products elicit differential *Diptericin* responses that are consistent across *Drosophila melanogaster* life stages. *D. melanogaster* adults **(A)** and 3rd instar larvae **(B)** were injected with 69.0 and 50.2 nl of non-activated (N) or activated (A) concentrated ES products, representing 138 and 100 infective juvenile equivalents, respectively. An equivalent volume of Ringer's buffer (R) served as a control. Flies and larvae were homogenized at a 6-h time point before RNA isolation, cDNA conversion, and transcript abundance quantification of the antimicrobial peptide *Diptericin* by qPCR. Fold change is relative to 0-h expression immediately following injection with each treatment and values represent data from three trials at two technical replicates per trial, where replicate measurements are drawn from the pooled cDNA of five flies or larvae (*p < 0.05, **p < 0.01).

The effects of concentrated ES products on the immune response of *Drosophila* were first examined in the context of the antimicrobial peptide (AMP) response. Imd and Toll pathway activity was assessed in flies by examining the expression of *Diptericin* and *Drosomycin*, respectively, following the injection of 69.0 nL of the highest concentration of ES products, a volume equivalent to the excretory/secretory output of 138 IJ. Expression was also assessed in larvae though with a lower injection volume of 50.2 nL, corresponding to ~100 IJ equivalents. Both adult flies and larvae were collected at a 6-h time point following ES injection, which was chosen to capture expression during peak Imd activity. *Drosomycin* transcript, as measured by qPCR, was not significantly altered by the injection of activated or non-activated ES products, and notably the products also failed to elicit a response at the 24-h time point known to correlate to peak Toll pathway activity (data not shown). Conversely, *Diptericin* was significantly upregulated by injection of non-activated ES products compared to the Ringer's buffer control injection. However, injection of activated ES products failed to increase *diptericin* expression above the Ringer's buffer control injection, suggesting the presence of suppressive or non-immunogenic components in activated ES (Figure 2). This pattern was observed in both adult flies and larvae, though on a slightly larger scale through all three treatments in larvae, possibly due to the primary immune organ, the fat body, being proportionally larger relative to body size in larvae. The immune response to a nematode infection minimally includes a strong Imd response, which is apparent through *Diptericin* expression, and the *H. bacteriophora* countermeasures to this activity are clearly capable of neutralizing the effect to levels associated with mere injury rather than infection.

### Excretory-Secretory Product-Based Differential *Diptericin* Responses Originate at or Prior to Transcriptional Activation

**Figure 3:**
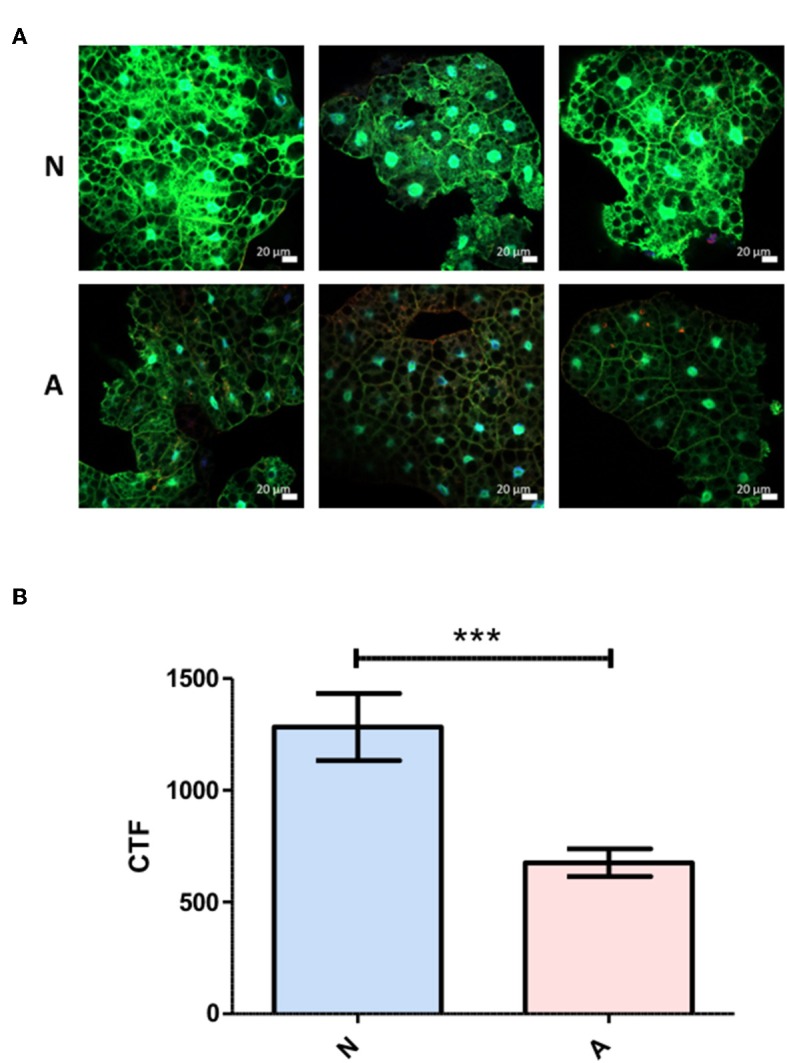
Differential *Diptericin* responses to ES products originate at or prior to transcriptional activation. **(A)** Larvae of a *Drosophila melanogaster* line carrying GFP under the control of the antimicrobial peptide *Diptericin* promoter were injected with 50.2 nl of non-activated (N) or activated (A) products. The fat body was extracted at a 6-h time point and imaged via confocal microscopy. One representative image from each of the three trials is shown for both treatments. **(B)** Corrected total fluorescence was assessed for isolated green channels with Image J software (***p < 0.001). Values were calculated for 10 images per treatment per trial.

To more precisely describe the effects of ES products on the regulation of *Diptericin*, larvae of a *Drosophila* line carrying GFP under the control of the *Diptericin* promoter were injected with ~100 IJ equivalents of either activated or non-activated products, and collected for observation at a 6-h time point. The fat body was dissected and imaged by confocal microscopy at 40x magnification. Fluorescence was clearly visible in all samples, though on average fat body samples that had been exposed to non-activated products were substantially brighter than those treated with activated ES products. This observation was confirmed by corrected total fluorescence (CTF) measurements of isolated green channels for each image (Figure 3). Because fluorescence is a measure of promoter activation, the specific interaction that mediates the differential responses to activated and non-activated ES products can be posited to take place either at or upstream of transcriptional activation. These measurements also confirm that the differences seen in *Diptericin* expression are mediated at least in part by cells of the fat body.

### Triple-Concentrated Activated *Heterorhabditis bacteriophora* Nematode ES Products Are Lethal to Adult *Drosophila melanogaster*

**Figure 4:**
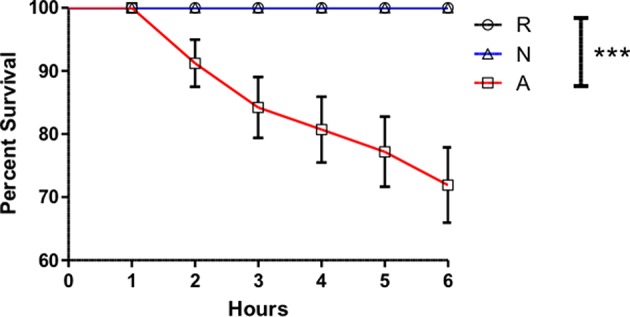
Activated *Heterorhabditis bacteriophora* nematode Excreted/Secreted (ES) products are lethal to adult *Drosophila melanogaster*. *Drosophila* adults were injected with 69.0 nl of Ringer's buffer (R) or 414 IJ-equivalent dose triple-concentrated ES products, either activated (A) or non-activated (N) and monitored for mortality every hour for 6 h, at which point injected populations typically stabilized and no additional deaths were observed up to a 24-h time point. Each curve is comprised of measurements for three trials of 10 male and 10 female flies (***p < 0.001).

**Figure 5:**
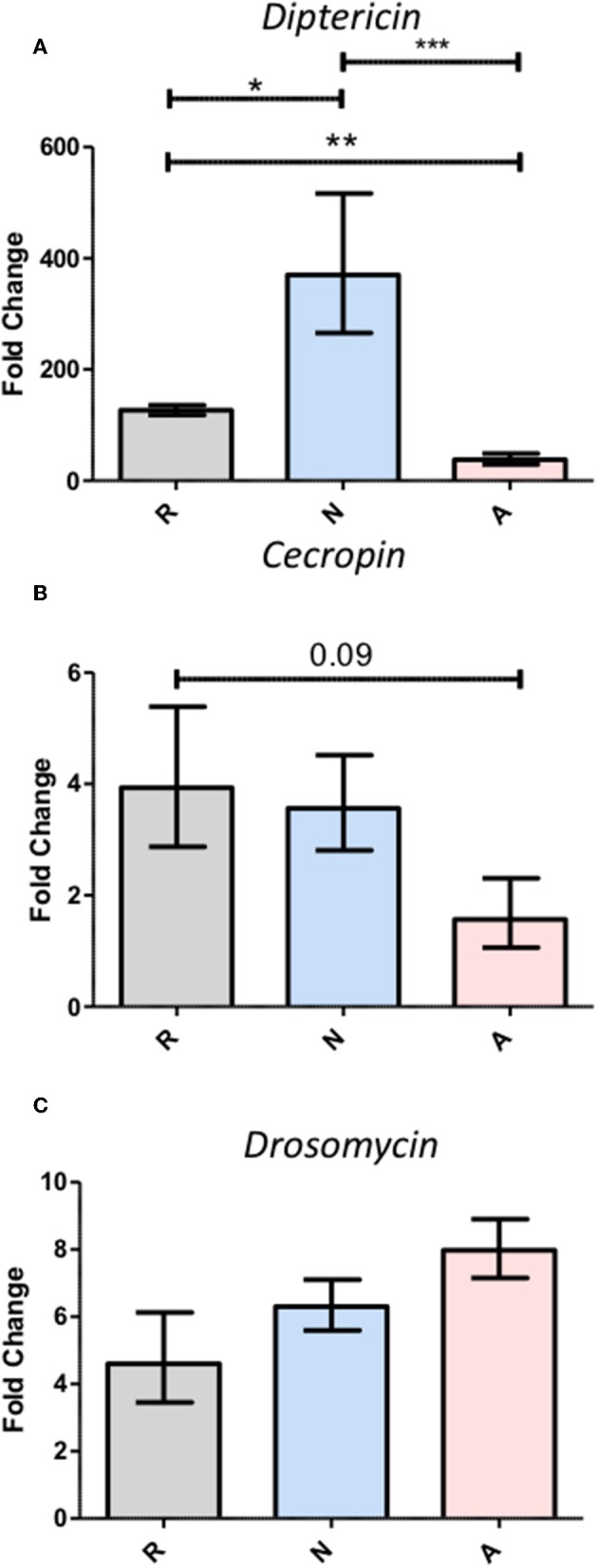
Triple-concentration of the *Heterorhabditis bacteriophora* nematode Excreted/Secreted (ES) products exacerbates *Diptericin* responses, but fails to elicit responses from other antimicrobial peptides. Adult *Drosophila melanogaster* were injected with 69.0 nl of triple-concentrated ES products prior to homogenization for RNA extraction at a 6-h time point. Gene expression normalized to *rp49* expression was assessed for the antimicrobial peptides *Diptericin* **(A)**, *Cecropin* **(B)**, and *Drosomycin* **(C)**. Bars represent fold change over the 0-h measurement for each treatment. Averages with standard error are shown for three trials performed in duplicate such that each trial produced two measurements for pooled cDNA from five flies (*p < 0.05, **p < 0.01, ***p < 0.001).

While the activated ES products clearly do not provoke as strong a *Diptericin* response as the non-activated products, the relative equivalence of the responses to Ringer's buffer and activated ES products makes it impossible to determine whether the activated nematode is secreting factors that suppress immunity or simply eliminating the production of factors that are immunogenic in the host. In an attempt to resolve this ambiguity, three separate batches of ES products produced with 200,000 IJs were concentrated together such that suppressive effects would be stronger, but the absence or masking of immunogenic compounds would not have compounding effects on *Diptericin* expression to limit upregulation below that evoked by a control injection. The increased potency of these products was immediately apparent, as injection of 414 IJ equivalents resulted in ~70% mortality over a period of 6 h (Figure 4). Flies that survived the injection at the 6-h time point were collected and *Diptericin* transcript levels measured. The 3x concentrated ES products significantly decreased the *Diptericin* response below that of the Ringer's buffer alone (Figure 5A), thus indicating that *H. bacteriophora* secretes factors capable of the specific suppression of *Diptericin* upregulation. The specificity of *Diptericin* suppression was examined by assessing the response of a second Imd-responsive AMP, *Cecropin*, as well as the Toll pathway AMP *Drosomycin* to the concentrated activated ES products. Injection of 414 IJ equivalents had no effect on either *Cecropin* (Figure 5B) or *Drosomycin* (Figure 5C) expression in adult flies.

### *Heterorhabditis bacteriophora* Nematode ES Products Promote Mortality Driven by Both Pathogenic and Non-pathogenic Bacteria

**Figure 6:**
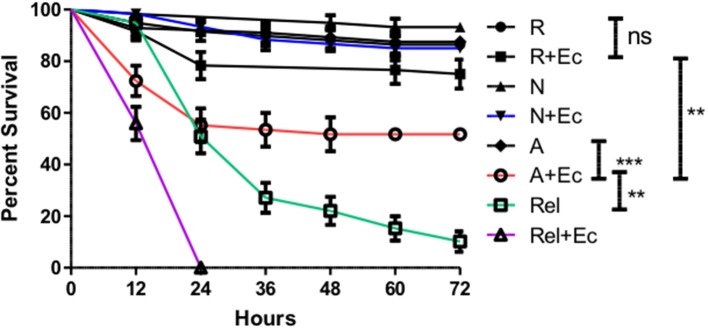
Co-injection of *Escherichia coli* with activated *Heterorhabditis bacteriophora* nematode Excreted/Secreted (ES) products results in fly mortality. Adult *Drosophila melanogaster* were injected with 69.0 nl of a 1:4 mixture of OD 3.0 *E. coli* (+Ec) and activated ES products (A), non-activated ES products (N), or Ringer's buffer (R). After mixing, solutions contained 310 IJ equivalents of ES products and 8 × 10^4^ CFUs of *E. coli* as applicable. *Relish* mutant flies (Rel) were also injected in order to compare the magnitude of ES-suppression to that of Immune deficiency pathway ablation. Survival was assessed every 12 h for a total of 72 h. Three trials were performed, each consisting of 10 male and 10 female flies per treatment. Where bars are omitted, standard error was negligible (ns *p* > 0.05, **p < 0.01, ***p < 0.001).

While the specific suppression of *Diptericin* is significant, this result does not allow conclusions about whether the ES products released by *H. bacteriophora* are sufficiently immunosuppressive to augment a bacterial infection. To explore this possibility, adult flies were co-injected with a high inoculum of *Escherichia coli* (8 × 10^4^ CFUs) and ~310 IJ equivalents of activated ES products, non-activated ES products, or an equivalent volume of Ringer's buffer. Tracking mortality every 12 h for a period of 72 h revealed that while Ringer's buffer or non-activated ES products co-injected with *E. coli* were not lethal to flies, the injection of activated products and *E. coli* together (A+Ec) resulted in ~50% mortality in the first 24 h (Figure 6). Notably, this reduced dose (310 vs. the lethal 414 IJ equivalents) of activated ES products no longer induces mortality, so the observed decrease in survival cannot be attributed to the previously noted lethality stemming from the products alone.

**Figure 7:**
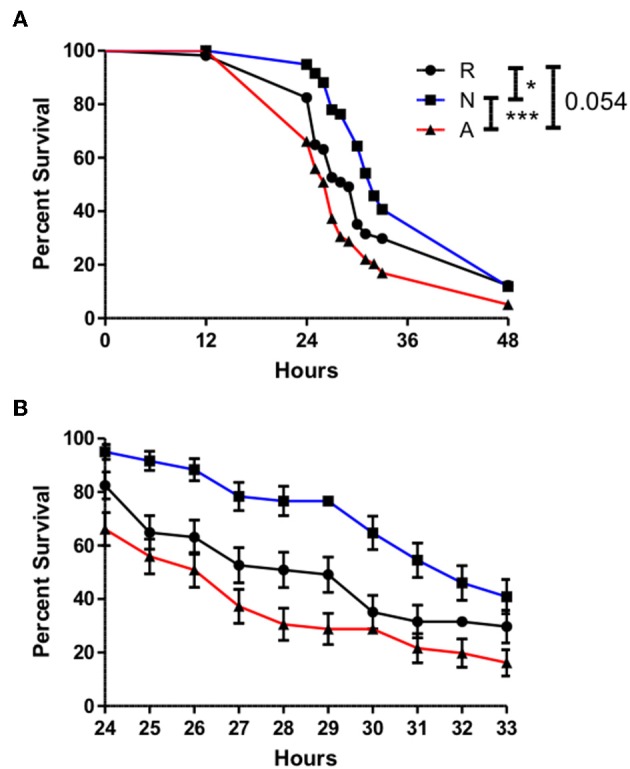
The onset of mortality evoked by *Photorhabdus luminescens* infection is significantly advanced by *Heterorhabditis bacteriophora* nematode Excreted/Secreted activated products, but delayed by non-activated products. Adult *Drosophila melanogaster* were injected with 69.0 nl of a 1:4 mixture of OD 0.4 *P. luminescens* bacteria and activated ES products (A), non-activated ES products (N), or Ringer's buffer (R), conveying 310 IJ equivalents of ES products and 50 CFUs of *Photorhabdus*. Survival was observed at 12 h and then every hour after 24 h until 33 h in order to capture the majority of mortality events at a higher resolution. A final time point was assessed at 48 h **(A)**. Mortality in injected flies between 24 and 36 h is shown in **(B)**. Curves depict average values collected over three trials of 20 flies, 10 males, and 10 females, per treatment (*p < 0.05, ***p < 0.001).

In the context of a natural *H. bacteriophora* infection, the bacteria of interest would be the natural symbiont of the nematode, *Photorhabdus luminescens*. The bacteria are released from the gut of the IJ shortly after entry into the hemolymph, and the possibility exists that the ES products may serve in part to prepare the hemolymph environment for a more successful infection by *P. luminescens*. This possibility was tested by similarly co-injecting adult flies with ~310 IJ equivalents of ES products or an equivalent volume of Ringer's buffer and 50 cells of *P. luminescens*. Time points were at 12 h, then every hour from 24 to 33 h, in order to capture the majority of mortality, and then once again at 48 h. Survival curves revealed a slightly protective effect imparted by the non-activated ES products relative to the control injection, and when compared to co-injections with activated ES products, the lethality produced by the activated ES products and *P. luminescens* was significantly different from and effected earlier than that produced by the non-activated ES product co-injections (Figure 7). Even at a sublethal dose, the ES products of *H. bacteriophora* are sufficiently immunosuppressive to negatively impact the AMP response and to enhance the virulence of a bacterial infection.

### *Photorhabdus luminescens* Proliferates More Rapidly in Adult *Drosophila* When Co-injected With Activated ES Products

**Figure 8:**
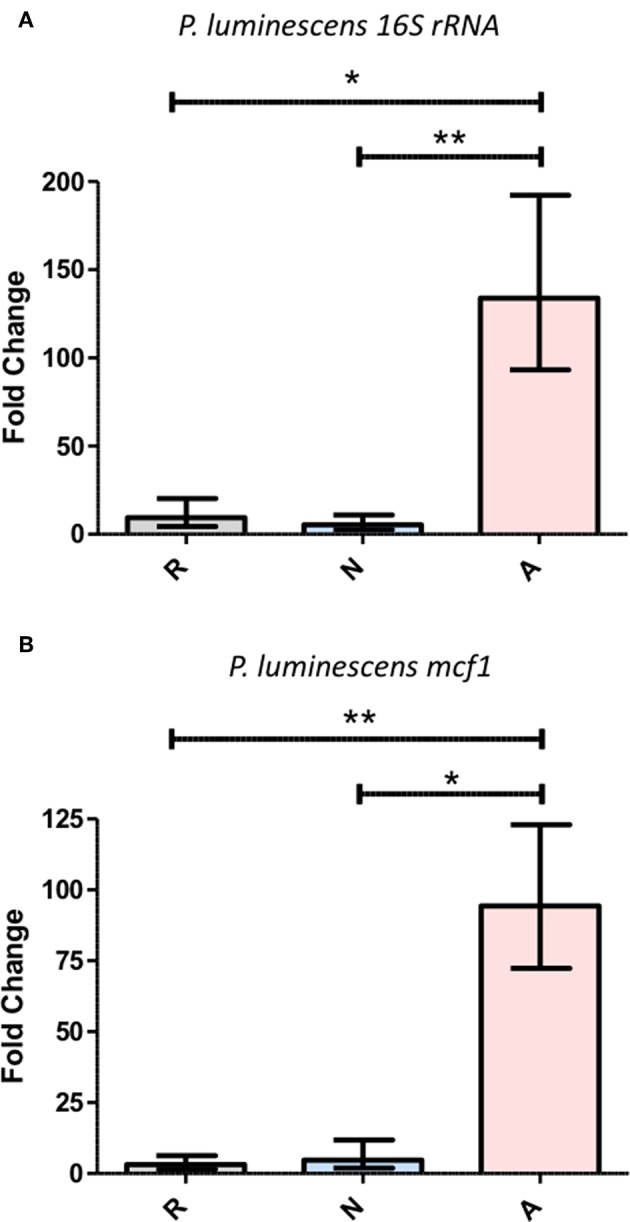
Activated *Heterorhabditis bacteriophora* ES products enable the rapid proliferation of *Photorhabdus luminescens* during the early phase of an infection. A 1:4 mixture of OD 0.4 *P. luminescens* and Ringer's buffer (R), non-activated ES products (N), or activated ES products (A) was injected into the thorax of adult *Drosophila*, which were then incubated for a period of 14 h. Following RNA extraction, gene expression was measured by RT-qPCR for *P. luminescens* 16S rRNA **(A)** as well as *mcf* **(B)**, both of which were normalized to *rp49*. Each graph shows fold change in expression between the 0 and 14 h time points. Three trials of two replicates with five flies per replicate were performed (*p < 0.05, **p < 0.01).

The delay in the onset of mortality for populations of flies co-injected with ES products and *P. luminescens* (Figure 7) demonstrates that this mortality initiated by *Photorhabdus* requires an accumulation of bacteria beyond the initial inoculum. To test whether the influence of activated ES products is capable of accelerating this accumulation, the co-injections of ES products and *Photorhabdus* were repeated under the same conditions and surviving flies were collected at a 14-h time point for the assessment of relative bacterial growth, as measured by RT-qPCR targeting the *P. luminescens* 16S rRNA and *mcf1* genes. Subsequent analysis of expression for both genes revealed that bacterial proliferation is significantly higher in the presence of activated ES products, which supported an ~100-fold transcript increase for each gene (Figure 8). Those treated with either Ringer's buffer or non-activated products showed increases between 3- and 10-fold for the same genes. This difference in bacterial survival and proliferation is therefore likely responsible for the ~12-h decrease in the time to mortality onset for flies co-injected with activated ES products as compared to those injected with non-activated products.

### *H. bacteriophora* ES Products Provoke a More Active Phagocytic Response

**Figure 9:**
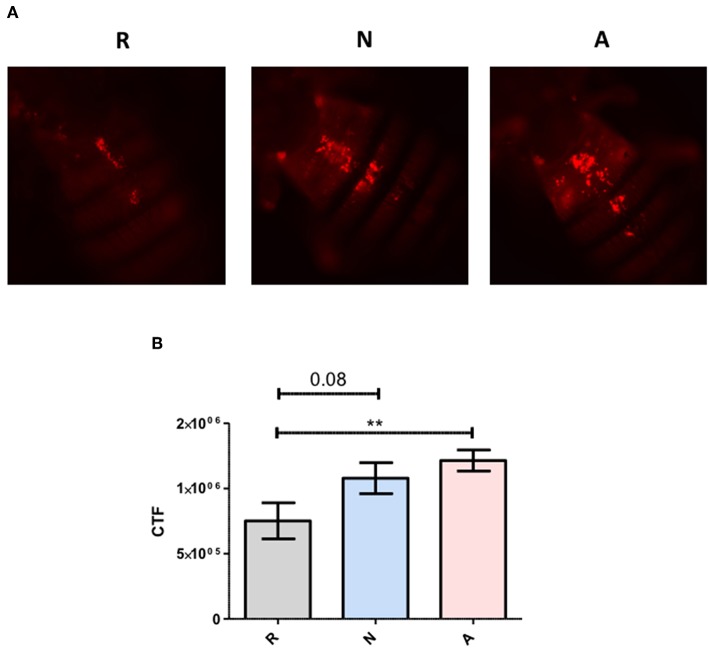
*Heterorhabditis bacteriophora* nematode activated Excreted/Secreted (ES) products provoke a stronger phagocytic response. Adult *Drosophila melanogaster* were injected with 69.0 nl of a 1:4 mixture of 4 mg/mL pHrodo *E. coli* conjugates and activated ES products (A), non-activated ES products (N), or Ringer's buffer (R). **(A)** Images were captured by fluorescence microscopy at 10x magnification and **(B)** the area associated with pericardial nephrocytes was analyzed with ImageJ software. Values are shown for measurements collected over three trials of three replicates each (**p < 0.01).

Another possible mechanism causing increased mortality when flies are challenged simultaneously with activated nematode ES products and bacteria is interference with the normal activity of phagocytic hemocytes. To determine whether this effect is also contributing to the enhanced success of bacteria in ES-treated flies, adult *D. melanogaster* were co-injected with ~310 IJ equivalents of ES products or an equal volume of Ringer's buffer and pHrodo *E. coli* conjugates that fluoresce when engulfed by a phagocyte. CTF measurements of images captured with fluorescence microscopy showed that phagocytic activity around the pericardium, where the highest degree of activity is observed, is significantly elevated in flies co-injected with activated ES products (Figure 9). The immunosuppressive effect of the ES products is not mediated by the phagocytic response, and may in fact provoke more phagocytic activity. Despite this compensatory phagocytic response, the effects on AMP production or other systems are still potent enough to enhance a bacterial infection.

## Discussion

With the entirety of the observed effects relying on the *in vitro* activation of IJs, the degree to which the collected ES products align with those of an *in vivo* infection should be addressed. For *Steinernema* species, activation has been shown to be influenced by host species, the age of the IJs being activated, the homogenate concentration used for activation, and the duration of exposure to host components (22, 23). These factors could similarly affect the activation of *H. bacteriophora*, which is able to infect lepidopterans, dipterans, coleopterans, hymenopterans, anoplurans, orthopterans, homopterans, and hemipterans to varying degrees of lifecycle completion (24). Each of these hosts may provoke a slightly different response from the IJs, possibly even a different assortment of ES products. Furthermore, *H. bacteriophora* is known to inhibit IJ development in a conspecific manner through a small-molecule pheromone termed C11 EA (25), indicating that the concentration of IJs could tune the activation state based on the ratio of suppressive conspecific signal to activating host signal. Laboratory propagation of the nematodes can also be a factor in that *Heterorhabditis* virulence can be affected by not only the number of generations that have been propagated in laboratory conditions, but also the number of IJs used to infect a host during each passage (26). It is therefore immediately crucial to concede that any collection of ES products from entomopathogens activated *in vitro* will likely not contain the ES products of the nematode in a universal sense, but rather a subset of products specific to a given activation and collection protocol. This fact does nothing however to diminish the practical or informative value of effects stemming from an isolation of ES products provided they accurately represent at least some subset of the virulence arsenal of the nematode. For the products used in this set of assays, our results demonstrate an effective activation through the emergence of a unique protein profile. The subsequent assays serve to identify functions of these proteins that are produced specifically in response to host hemolymph.

The first effect of the ES products to be observed was the capacity of non-activated ES products to provoke higher expression of the antimicrobial peptide gene *Diptericin* following injection into adult *Drosophila*. This gene was selected by virtue of its role as a readout of the Imd pathway, for which the best described function is the production of antimicrobial peptides in response to Gram-negative bacteria (27). The Imd pathway is relevant because the bacterial symbiont of *H. bacteriophora, Photorhabdus luminescens*, is a Gram-negative bacterium, but also because of the pathway's association with septic injury in general (28), which would imply that Imd activation could also occur during penetration of the nematode into the cuticle. Additionally, the Imd pathway appears to have a larger role in inflammation and immunity based on its contribution to the viral response (29, 30), which further asserts that Gram-negative bacterial pathogen-associated molecular patterns (PAMPs) are not its sole activating inputs. The initial expression changes observed here imply that basally expressed components of non-activated nematode secretions are also capable of directly or indirectly promoting Imd activity, and possibly in a specific manner. This is supported by the data shown here, as expression of *Diptericin* is believed to be regulated solely by the Imd pathway as opposed to having a regulatory mode like that of *Attacin*, which is thought to receive inputs from both the Toll and Imd pathways (31). After demonstrating the immunogenicity of non-activated products in adults, the effect was confirmed in whole larvae, the stage more commonly associated with IJ infection, as well as specifically in the fat body. Importantly, the latter provides the additional information that the immunogenic effect of the non-activated products involves a systemic response from the fat body, a crucial distinction given that *Diptericin* can be expressed locally in sections of the digestive tract, specifically the proventriculus and midgut (32). Results from the Dpt-GFP assay also provide an assurance that differences stem from activity taking place at or before transcription, but additional work will be required to specify a mechanistic point of interference beyond that simple binary.

To determine whether the activated products simply lacked immunogenicity or were instead carrying out targeted suppression, the products were triple-concentrated by combining the secretions of three separate activations of 200,000 IJs. These more concentrated products were lethal through early timepoints following injection into adult flies, although less so than the secretions of *Steinernema carpocapsae* (10). This is consistent with previous findings regarding the *in vivo* virulence of axenic IJs of these two species (33). When the effect on *Diptericin* expression was reassessed with this higher 414 IJ equivalent dose, the upregulation induced by the activated products was significantly lower than that of the Ringer's buffer injection, while the non-activated products continued to display consistent immunogenicity. Because a loss of immunogenicity would do nothing to eliminate Imd activity induced by the vehicle control, the 414 IJ equivalent injections reveal targeted immune suppression by the activated ES products. The argument could be made here that *H. bacteriophora* might generate antibiotic compounds during an infection and that these are reducing the population of Imd-activating microbes introduced by the injection. While *P. luminescens* is known to produce antibiotics (34), no such activity has been attributed to *H. bacteriophora*, and this scenario would be in stark contrast to the results of the bacterial co-injection survival assays, especially that of *E. coli*. If the ES products contain antibiotics, they should be strongly protective after co-injection. The suppressive capacity of the ES products was then tested for two other antimicrobial peptide genes, *Cecropin* and *Drosomycin*, which are regulated by the Imd and Toll pathways, respectively. Neither of these genes showed any significant differences between the three treatments, indicating that the transcriptional suppression observed in the case of *Diptericin* may be specific for that gene, though other gene products may be affected at different levels of host-parasite interactions. An infection by the filarial nematode *Brugia pahangi* can be inhibited by Cecropins (35), but if this is also the case for *Heterorhabditis, H. bacteriophora* has mediated this threat through the synthesis of a proteinase capable of degrading Cecropins (36), effectively eliminating the pressure to suppress *Cecropin* transcriptionally. The absence of a *Drosomycin* response may simply be the product of irrelevance given that neither *S. carpocapsae* nor *H. bacteriophora* nematodes induce *Drosomycin* expression in *Drosophila* larvae if the nematodes are axenic (3, 37). Generally though, the lack of activity on other antimicrobial peptide genes does at least demonstrate that the suppression of *Diptericin* is a more subtle, targeted effect than broad interference with immune gene transcription.

Having demonstrated that *H. bacteriophora* IJs respond to a host by secreting a unique set of proteins possessing immunomodulatory activity, the ES products were then tested for their contribution to infection outcome, particularly one instigated by Gram-negative bacteria due to their susceptibility to Imd outputs. Flies that were injected with activated ES products and non-pathogenic *E. coli* (38) displayed significantly increased mortality as compared to controls, which showed that this dose of *E. coli* is not lethal by itself. Mortality occurred predominantly within 24 h of injection, after which point the rate of mortality declined sharply, implying that the active proteins in the ES products are degraded or otherwise buffered by the fly at later time points. *Relish* mutant flies were also injected with *E. coli* or Ringer's buffer in order to serve as a comparison for the magnitude of suppression. *Relish* is the terminal transcription factor in the Imd pathway and accordingly, these flies are highly susceptible to infection by Gram-negative bacteria (39). If these flies and those treated with activated ES products are equally susceptible, this would imply a nearly complete suppression of the Imd pathway by activated ES products. Interestingly, the trajectory of the *E. coli* and activated ES products co-injection survival curve does most closely resemble the *Relish* mutant *E. coli* injection curve at the earliest time point, but the treatments then diverge. Generally, this effect is illustrative of the immunosuppressive capacities of the ES products, but this is still more or less inconsequential in a natural infection unless the ES products can also support the *H. bacteriophora* symbiont *P. luminescens*. The Imd pathway has been previously implicated in the immune response to *P. luminescens* in that *Diptericin* is strongly upregulated following bacterial injection, and the avirulent *phoP* strain of *Photorhabdus* is restored to full pathogenicity in Imd pathway mutants (40). The *Diptericin*-specific suppression facilitated by the activated ES products is thus likely relevant to the survival of *Photorhabdus* in *Drosophila*. Co-injections with ES products were repeated with a far less concentrated, ~50 CFU inoculum of *P. luminescens*, which is representative of the average bacterial load of an *H. bacteriophora* IJ (2). The co-injection of activated ES products led to a significantly earlier onset of mortality as compared to non-activated products while the latter also displayed a slightly protective effect as compared to Ringer's buffer, potentially due to the elevated induction of *Diptericin* expression. Populations of injected flies were also stable until after the 12-h time point, reaffirming the specific role of the bacteria in the mortality of co-injected flies. Furthermore, this delay compared to the *E. coli* co-injections implies that the injected *Photorhabdus* needed to replicate substantially to achieve a lethal concentration. Other findings have shown that the population responsible for eventual septicemia in an insect originates from a small subpopulation that is resistant to antimicrobial peptides (41), so part of the role of nematode ES products might be to bolster this subpopulation as much as possible. Our data support this idea in that relative *Photorhabdus* abundance at a 14-h time point, just after the onset of mortality, was an order of magnitude higher in flies co-injected with activated ES products. Other time points could be examined to more fully enunciate the relationship between the presence of activated ES products and *Photorhabdus* growth kinetics, but this time point was considered the most critical and sufficient for demonstrating the practical capacity of ES-based suppression. Furthermore, this system could also eventually be used to examine the interplay between *Heterorhabditis* and *Photorhabdus* virulence factors with regard to AMP suppression through different phases of the infection.

Finally, to eliminate the possibility that survival differences were stemming from the phagocytic response, *H. bacteriophora* ES products were co-injected with pHrodo *E. coli* conjugates to measure overall phagocytic activity. Activated products were found to significantly increase ingestion of the conjugates, but this increase in phagocytosis was clearly unable to promote survival during infection, which is consistent with findings that knock-down of the phagocytic receptor Nimrod C1 has no effect on the survival of *Drosophila* during an infection by symbiotic *H. bacteriophora* (42). Although this is not a comprehensive assessment of the cellular response or related immune mechanisms, our future work will focus on analyzing the effects of the ES products on several other processes including melanization, encapsulation, and clot formation.

Much of the immune response has been left uninvestigated by this set of assays, in particular the immune response specifically against the nematode, but the pattern observed here reveals a cohesive image of specific immune gene suppression that could play a crucial role in the infection process. Together, the conclusions of this work show that *H. bacteriophora* secretes a unique protein profile in response to a host, this collection of proteins suppresses the expression of the antimicrobial peptide-encoding gene *Diptericin*, and suggest that the suppressive capacity of the secreted products allows a small population of *P. luminescens* to propagate and overwhelm a host more quickly. This represents a fundamental component of nemato-bacterial bipartite virulence and provides a strong justification for exploring the individual components of the secreted products produced by the nematode in order to identify specific immunosuppressive proteins that could be employed in a variety of applications. The interaction of these individual proteins with host immune mediators can then be observed in the context of the effects described here, with the aim of providing a mechanistic explanation for *Heterorhabditis*-based immunosuppression. Given the wealth of molecular components that could be targeted to interfere with Imd responses, even outside the signaling components of the Imd pathway, it would be premature to suggest a mechanism from the effects observed here, but potential avenues of research can be suggested. One well-supported field of inquiry would be to examine the ability of these ES products to interfere with eicosanoid production. In insects, eicosanoid production relies on the ability of phospholipase A_2_ (PLA_2_) to synthesize eicosanoid precursor lipids like arachidonic acid (AA), and interference with this pathway can have strong immunosuppressive effects based on the role of eicosanoids in the regulation of cellular and humoral responses, including *Diptericin* expression through the Imd pathway (31, 43). *Photorhabdus* is known to inhibit PLA_2_ (44), but a variety of parasitic nematodes also secrete proteins that could similarly interfere with eicosanoid synthesis through their ability to bind fatty acids, including arachidonic acid, which could sequester necessary eicosanoid precursors (45). Similar proteins have also been found in the ES products of *Steinernema carpocapsae* (10) and the transcriptome of activated *H. bacteriophora* (14). Interference with this pathway would be consistent with the findings presented here and an efficient way for the parasite to simultaneously suppress multiple immune responses.

## Data Availability Statement

The datasets generated for this study are available on request to the corresponding author.

## Author Contributions

EK conceived and designed the experiments, performed the experiments, analyzed the data, wrote the paper, prepared the figures, and reviewed drafts of the paper. JH, DO'H, and IE conceived and designed the experiments, supervised the project, reviewed drafts of the paper, and prepared the manuscript.

### Conflict of Interest

The authors declare that the research was conducted in the absence of any commercial or financial relationships that could be construed as a potential conflict of interest.
